# A bacterial effector protein targets plant ferredoxin-NADP+ reductase to promote infection

**DOI:** 10.1371/journal.ppat.1013664

**Published:** 2025-10-30

**Authors:** Lihaitian Wang, Xiaoli Liu, Feng Yu, Wenxuan Pu, Xiaoxu Li, Dousheng Wu

**Affiliations:** 1 Hunan Key Laboratory of Plant Functional Genomics and Developmental Regulation, Hunan Research Center of the Basic Discipline for Cell Signaling, College of Biology, Hunan University, Changsha, China; 2 Research Institute of HNU in Chongqing, Chongqing, China; 3 Beijing Life Science Academy, Beijing, China; 4 Technology Center, China Tobacco Hunan Industrial Co., Ltd., Changsha, China; University of Florida Institute of Food and Agricultural Sciences, UNITED STATES OF AMERICA

## Abstract

Pathogenic bacteria utilize a type III secretion system to translocate effector proteins into plant cells, where they inhibit plant immunity or interfere with normal cellular functions to facilitate infection. Whether and how pathogen effectors manipulate plant adenosine 5’-triphosphate (ATP) to facilitate infection remains largely unknown. In this work, we show that an effector protein, RipAF1, from the plant pathogen *Ralstonia solanacearum* suppresses flg22-induced immune activation and contributes to virulence. RipAF1 physically interacts with plant ferredoxin-NADP^+^ reductase (FNR), which is involved in NADPH and ATP production, in chloroplast. Transient expression of FNR leads to increased ATP accumulation and resistance against *R. solanacearum*, while co-expression of FNR with RipAF1 significantly reduced ATP levels. We further show that exogenous application of ATP enhances plant resistance to *R. solanacearum* infection. Our findings indicate a key role of ATP in plant resistance against *R. solanacearum,* and elucidate a bacterial virulence strategy wherein pathogenicity is enhanced through targeted modification of host ATP homeostasis via bacterial effector proteins.

## Author summary

Immune responses are highly energy-consuming processes. ATP, the universal energy currency for cellular processes, is essential for the immune responses that plants and animals need to defend against pathogen attacks. Extracellular ATP can also function as damage-associated molecular pattern to trigger defense reaction. Type III secretion systems and their secreted effector proteins are key virulence factors for bacterial pathogens. These effector proteins have long been thought to primarily directly suppress host immunity. However, emerging evidence indicates that pathogen effector proteins can also interfere with other cellular processes, such as hormone metabolism and protein degradation, to indirectly inhibit plant immunity.

In this study, we discovered that RipAF1, an effector protein from the broad-host plant pathogen *Ralstonia solanacearum*, interacts with ferredoxin-NADP⁺ reductase (FNR) in the host. FNR is a flavoenzyme involved in ATP production, and RipAF1 can reduce the host’s ATP content. Furthermore, we demonstrated that ATP pre-treatment can enhance plant resistance to *R. solanacearum* infection. Our research not only reveals the positive role of ATP in plant defense against this bacterial pathogen but also proposes a new molecular mechanism by which pathogens promote infection through interference with host energy metabolism.

## Introduction

Bacterial pathogens cause a variety of plant diseases on agriculturally important crops and thus are a major threat to food security. To successfully infect host plants, microbial pathogens employ virulence factors that promote invasion under appropriate environmental conditions. The type III secretion system (T3SS) is a key virulence factor utilized by most gram-negative plant pathogenic bacteria [1]. The needle-like T3SS directly injects effector proteins into plant cells, allowing the manipulation of plant cellular processes to the benefit of the pathogen. One major molecular function of pathogen effectors is the inhibition of plant innate immunity, which is activated upon recognition of pathogen-associated molecular patterns (PAMPs), such as bacterial elongation factor peptide elf18 or the immunogenic peptide flg22 derived from bacterial flagellin, by plant pattern recognition receptors (PRRs) [2,3]. Some effectors are recognized by nucleotide-binding-/leucine-rich-repeat receptors, which are key components of effector-triggered immunity (ETI) [4,5]. In addition to the suppression of plant immunity, pathogen effectors also interfere with other cellular processes including phytohormone signaling, gene expression, protein degradation and metabolism [2,6,7]. For example, transcription activator-like effectors from *Xanthomonas* spp. and *Ralstonia solanacearum* bind to specific DNA sequences and work as transcription activators regulating the expression of target genes in host cells [8]. Given the complexity of a natural infection, it is likely that pathogen effectors regulate many more cellular processes to facilitate invasion.

Adenosine 5’-triphosphate (ATP) is a universal energy source for all living organisms. ATP functions as an important substrate and co-factor in various intracellular biochemical processes and is typically maintained at millimolar levels inside the cell under normal conditions [9]. In response to wounding or environmental stimuli, ATP can be released from the cytosol to the extracellular space where it is recognized as a damage-associated molecular pattern (DAMP) [10]. Extracellular ATP (eATP) signaling has been shown to play a variety of roles in plant growth, biotic and abiotic stress responses [11]. The L-type lectin receptor-like kinase DORN1 functions as a receptor for eATP in plants [12]. Perception of eATP results in the production of reactive oxygen species (ROS), the elevation of cytoplasmic calcium and the activation of mitogen-activated protein kinases (MAPKs) [13], which resemble plant immune responses. DORN1 positively regulates plant defense response against the bacterial pathogen *Pseudomonas syringae* and the oomycete pathogens *Phytophthora infestans* and *Phytophthora brassicae* [14–17]. Treatment with ATP promotes defense-related gene expression and enhances plant resistance against the fungus *Botrytis cinerea* [18,19]. ATP treatment also induces stomatal immunity against *P. syringae* [13]. Despite that ATP is widely involved in plant resistance against pathogens, whether and how pathogens manipulate host ATP levels to their own benefit is currently unknown.

*R. solanacearum* is an economically and scientifically important bacterial plant pathogen that causes bacterial wilt disease on more than 250 plant species, including *Solanum lycopersicum*, *Capsicum annuum*, *Nicotiana benthamiana* and *Nicotiana tabacum* [20,21]. *R. solanacearum* enters plants through the roots and colonizes the water-transporting xylem vessels, ultimately causing wilting of the entire plants [22]. Like other Gram-negative bacterial pathogens, the main virulence determinant of *R. solanacearum* is the T3SS and its secreted effectors [22]. Individual strains encode 45–76 different effector proteins, and this relatively large and varied effector repertoire might be one of the factors that determines the wide host range of *R. solanacearum* [23]. *R. solanacearum* uses its effectors to suppress plant immunity [23,24]. Several recent studies highlighted that manipulation of host metabolism is a major function of *R. solanacearum* effectors [25–27]. RipAF1 is one of the effector proteins that is conserved in phylotype I, II and III *R. solanacearum* strains, and belongs to the HopF effector family [28]. A previous competitive index assay revealed a potential role of RipAF1 in bacterial fitness on eggplant [29]. RipAF1 has also been shown to localize to the cell periphery and nucleus, and interferes with flg22-triggered ROS production [30,31]. Furthermore, RipAF1 mediates the antagonistic crosstalk between the jasmonic acid (JA) and salicylic acid (SA) signaling pathways through the ADP-ribosylation of Fibrillin 1 (FBN1) [32]. In this work, we found that RipAF1 contributes significantly to *R. solanacearum* virulence in *N. benthamiana*, tobacco and *Arabidopsis thaliana*. We show that RipAF1 interacts with plant ferredoxin-NADP^+^ reductase (FNR), which catalyzes the conversion of NADP^+^ to NADPH and is involved in ATP production [33]. Over-expression of RipAF1 decreases host NADPH and ATP levels, promoting infection. Therefore, our results reveal a positive role of FNR in bacterial wilt resistance and a virulence mechanism used by pathogens to facilitate infection.

## Results

### RipAF1 contributes to the virulence of *R. solanacearum*

**Fig 1:**
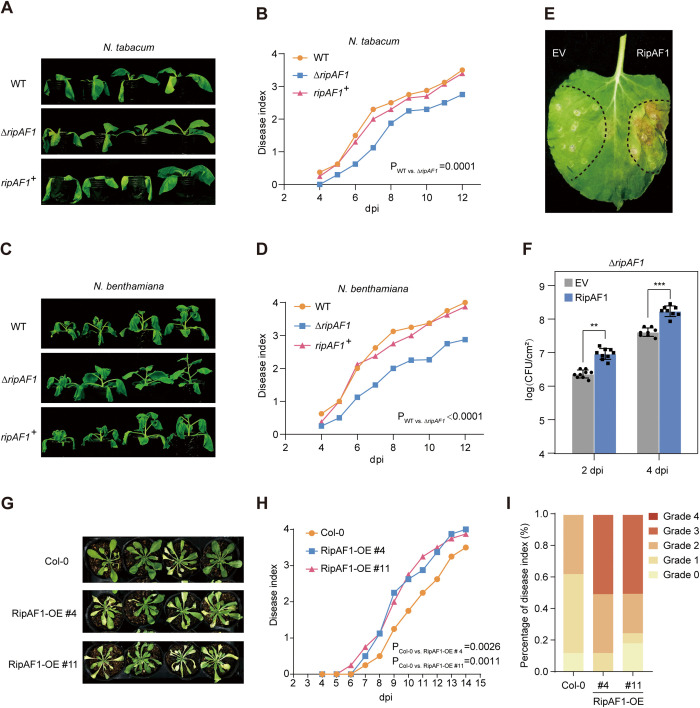
RipAF1 contributes to *R. solanacearum* virulence. **(A-D)** Soil-drenching inoculation assays in *N. tabacum* (**A** and **B**) and *N. benthamiana* (**C** and **D**) with the wild-type strain CQPS-1 (WT), the RipAF1 mutant strain *∆ripAF1* and the RipAF1 complementation strain *ripAF1*^*+*^. Pictures of bacterial wilt symptoms (**A** and **C**) were taken at 12 days post inoculation. Disease index (**B** and **D**) was rated daily using a scale of 0 to 4. Each value represents the mean disease index (n = 8). Statistics analysis was performed with Dunnett’s multiple comparison test. The experiment was repeated three times with a similar result. **(E and F)** Growth of *R. solanacearum* in *N. benthamiana* leaf after transient expression of RipAF1. *N. benthamiana* leaf was first infiltrated with *A. tumefaciens* expressing GFP (EV) or RipAF1. Two days later, leaves were inoculated with *∆ripAF1* at 10^5^ CFU/ml. Leaf symptoms (**E**) were taken at 8 days post inoculation. Bacterial growth (**F**) was quantified at 2- and 4- days post inoculation, respectively. Data shown indicate mean ± SD (n = 9); **p < 0.01; ***p < 0.001 (Student’s *t*-tes*t*); solid dots, individual biological replicates. (**G** to **I**) Soil-drenching inoculation assay in *RipAF1* over-expressing *A. thaliana* plants. Four-week-old *A. thaliana* plants were soil-drench inoculated with a suspension of *R. solanacearum* (OD_600_ = 0.1) at 10 mL per plant. **(G)** Pictures of bacterial wilt symptoms were taken at 10 days post inoculation. **(H)** Disease index was recorded daily. Each value represents the mean disease index (n = 18). Statistics analysis was performed with Dunnett’s multiple comparison test. (**I**) indicates the disease index distribution at 9 dpi. Grade represents the 0 – 4 disease index (0, no leaf wilted; 1, 1% to 25% leaves wilted; 2, 26% to 50% leaves wilted; 3, 51% to 75% leaves wilted; 4, 76% to 100% leaves wilted). The experiment was repeated three times with a similar result.

To determine the contribution of RipAF1 to *R. solanacearum* virulence, we generated a *∆ripAF1* knockout mutant strain in the background of CQPS-1 (wild-type strain, WT) (S1A and S1C Fig), which was recently isolated from diseased tobacco plants [34]. Upon soil-drenching inoculation, the *∆ripAF1* mutant showed significantly delayed bacterial wilt disease progression on both tobacco (Fig 1A and 1B) and *N. benthamiana* (Fig 1C and 1D). Complementation of *RipAF1* into the *∆ripAF1* mutant almost completely rescued the virulence attenuation of the mutant strain (Figs 1A–1D, S1B, and S1D), indicating that RipAF1 contributes to *R. solanacearum* virulence. To further determine the impact of RipAF1 on bacterial replication *in planta*, we expressed *RipAF1* in *N. benthamiana* using *Agrobacterium tumefaciens*-mediated transient gene expression, followed by leaf infiltration with *R. solanacearum*, as previously described [35]. Since the WT strain contains RipAF1, which might partially overlap with RipAF1 over-expression in *N. benthamiana*, we used *∆ripAF1* for the *in planta* bacterial growth assay. The *∆ripAF1* strain carries a tetracycline-resistance gene, allowing for discrimination between *R. solanacearum* and *A. tumefaciens* on tetracycline plates. Compared to the control tissue expressing GREEN FLUORESCENT PROTEIN (GFP) (Empty vector, EV), expression of RipAF1 followed by *R. solanacearum* inoculation resulted in more severe disease symptoms and higher bacteria titer (Fig 1E and 1F). *R. solanacearum* infects a wide range of plants, including *A. thaliana*. Consequently, the *R. solanacearum*-*A. thaliana* pathosystem has been widely used to study bacterial-host interactions [24,36,37]. To further evaluate the virulence function of RipAF1 in *A. thaliana*, we generated transgenic *RipAF1* over-expression *A. thaliana* plants. Two independent *RipAF1*-overexpressing transgenic lines were inoculated with the WT *R. solanacearum*. *RipAF1*-overexpressing plants exhibited significantly enhanced disease susceptibility compared to wild-type Col-0 plants (Fig 1G-1I), providing compelling genetic evidence for RipAF1’s crucial role in bacterial pathogenesis. These results indicate that the conserved effector protein RipAF1 from *R. solanacearum* promotes bacterial virulence.

### RipAF1 suppresses flg22-triggered immune responses

**Fig 2:**
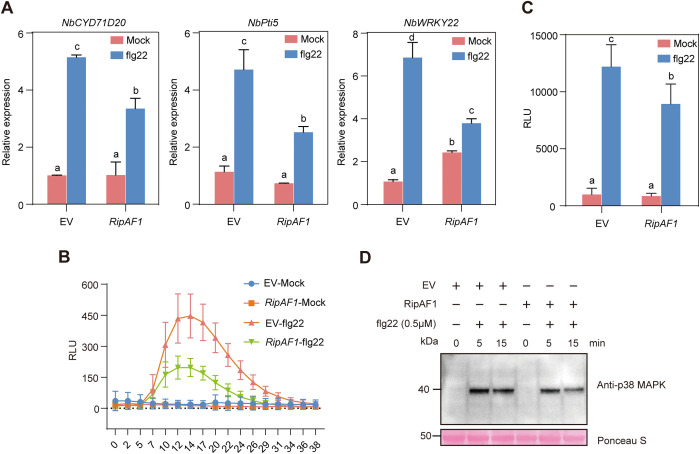
RipAF1 suppresses flg22-triggered immune responses. **(A)** Transient expression of *RipAF1* in *N. benthamiana* inhibits flg22-induced immune gene expression. *N. benthamiana* leaf was infiltrated with *A. tumefaciens* expressing GFP (EV) or RipAF1. Two days later, leaves were infiltrated with water (mock) or flg22 (100 nM). Leaves were harvested at 1 hour post infiltration for RNA extraction and qRT-PCR analysis. Data shown indicate mean ± SD (n = 3). **(B and C)** RipAF1 suppresses flg22-induced ROS burst in *N. benthamiana*. Leaf discs expressing GFP or RipAF1 were incubated in sterile water overnight and then treated with flg22 (500 nM). The luminescence was measured immediately in a 40 min time-scale. (**B**) shows the dynamic relative light unit (RLU) change. (**C**) shows the total RLU of different treated samples. Data shown indicate mean ± SD (n = 5). **(D)** Transient expression of RipAF1 suppresses flg22-induced MAPK activation. MAPK activation was detected at depicted time points by immunoblotting with an anti-pMAPK antibody. Protein loading was shown by Ponceau S staining. Different letters indicate statistical difference between different samples (one-way ANOVA, p < 0.05).

Key to the disease-promoting function of many type III effectors is their ability to suppress PAMP-triggered immunity [38,39]. Since RipAF1 facilitates bacterial replication and disease development on tobacco and *N. benthamiana* (Fig 1), we sought to determine if RipAF1 suppresses PAMP-triggered immunity. We first quantified the relative mRNA level of *NbCYP71D20*, *NbPit5*, and *NbWRKY22*, three flg22 responsive marker genes in *N. benthamiana* [40–42], after flg22 treatment in the absence or presence of RipAF1. Compared to the GFP control, expression of RipAF1 attenuated the flg22-induced activation of *NbCYP71D20*, *NbPit5*, and *NbWRKY22* (Fig 2A). A recent work revealed a role of RipAF1 from the reference strain GMI1000 in delaying ROS production when transiently expressed in *N. benthamiana* leaves [31]. We examined whether RipAF1 from CQPS-1 can inhibit flg22-induced ROS production and found that RipAF1 slightly but consistently reduced the flg22-triggered ROS burst compared to the GFP control (Fig 2B), although there was no statistically significant difference between GFP- and RipAF1-expressing leaves due to large variation among individual technical replicates. However, expression of RipAF1 significantly reduced the total ROS production (Fig 2C). This observation is different from the previous finding that RipAF1 did not reduce the total ROS production [31], possibly due to the different time scale used for ROS measurement. We further compared flg22-induced activation of MAPKs in *N. benthamiana*. The result showed that expression of RipAF1 has a weak inhibitory effect on flg22-triggered MAPK activation (Fig 2D). Taken together, these results indicate that RipAF1 suppresses flg22-triggered immune responses.

### RipAF1 interacts with plant ferredoxin-NADP+ reductase

**Fig 3:**
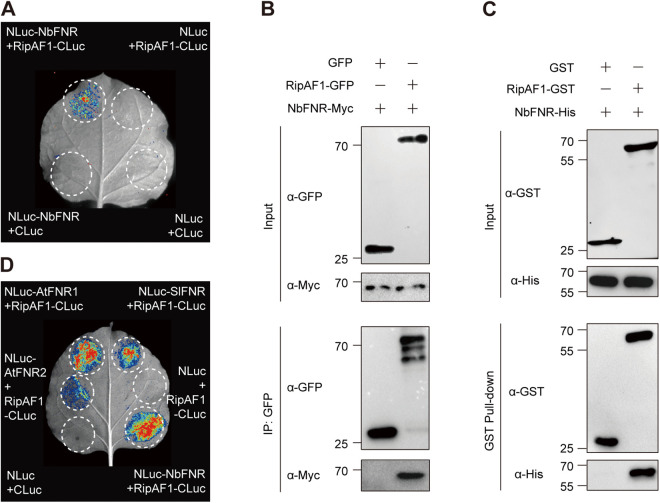
RipAF1 interacts with ferredoxin-NADP+ reductase. **(A)** Interaction between RipAF1 and *N. benthamiana* ferredoxin-NADP^+^ reductase (NbFNR) by split-LUC assay. *N. benthamiana* leaves were co-infiltrated with *A. tumefaciens* expressing NbFNR-nLUC and RipAF1-cLUC. Empty nLUC and cLUC were used as negative controls. The luciferase activity was detected by the imaging system at 36 to 48 hours post infiltration. **(B)** Interaction between RipAF1 and NbFNR by co-immunoprecipitation assay. RipAF1-GFP and NbFNR-Myc were co-expressed in *N. benthamiana* leaves and samples were harvested at 3 days post inoculation. Total protein was extracted and immunoprecipitated with anti-GFP Magnetic beads. Immunoblots were performed with anti-GFP and anti-Myc antibodies. **(C)** Association between RipAF1 and NbFNR by *in vitro* pull-down assay. GST or RipAF1-GST proteins were co-incubated with NbFNR-His protein. Immunoblots were performed with anti-GST and anti-His antibodies. **(D)** Interaction between RipAF1 and FNR from tomato and *A. thaliana* by split-LUC assay. Indicated constructs were co-expressed in *N. benthamiana* leaves. The luciferase activity was detected at 36 to 48 hours post infiltration. The experiment was repeated three times with similar results.

Next, we sought to identify the target protein(s) of RipAF1 in host cells. To do this, we transiently expressed C-terminal GFP-tagged RipAF1 (RipAF1-GFP) in *N. benthamiana*, which is a natural host of *R. solanacearum*, to perform immunoprecipitation (IP) using anti-GFP Trap beads and analyze interacting proteins using liquid chromatography followed by tandem mass-spectrometry (LC-MS/MS). Free GFP was used as a negative control to exclude nonspecific binding. After elimination of proteins found in the GFP samples, 94 candidate interactors were identified (S1 Table). Ferredoxin-NADP^+^ reductase (FNR) was identified as a candidate interacting protein of RipAF1 with high confidence (S2 Fig). To confirm the interaction between NbFNR and RipAF1 *in planta*, we cloned NbFNR into N-LUC and RipAF1 into C-LUC, respectively, and performed split-luciferase (split-LUC) assay. The high luciferase activity in NbFNR and RipAF1 co-expressed area indicates interaction between these two proteins (Fig 3A). The interaction was further validated by Co-IP of RipAF1 tagged with GFP and NbFNR tagged with Myc transiently co-expressed in *N. benthamiana* (Fig 3B). We also performed *in vitro* pull-down assays to confirm the direct interaction between NbFNR and RipAF1. RipAF1-GST and NbFNR-His fusion proteins were first expressed in *E. coli* and then purified (S3 Fig). NbFNR-His was pulled down by RipAF1-GST, but not by GST control, indicating the direct interaction between NbFNR and RipAF1 (Fig 3C). Since *R. solanacearum* has a wide range of host plants, FNR is conserved among different hosts by blast analysis (S4 Fig). To further determine whether RipAF1 interacts with FNR homologs from other hosts, we selected one FNR copy in tomato (*Solanum lycopersicum*, *SlFNR*) and two homologs in the model plant *A. thaliana* (*AtFNR1*, *AtFNR2*). Split-LUC assay showed that RipAF1 strongly associates with SlFNR and AtFNR1, the interaction between RipAF1 and AtFNR2 was weaker (Fig 3D). Taken together, RipAF1 directly targets FNR in multiple host plants.

### RipAF1 and NbFNR co-localize and interact in the chloroplast

**Fig 4:**
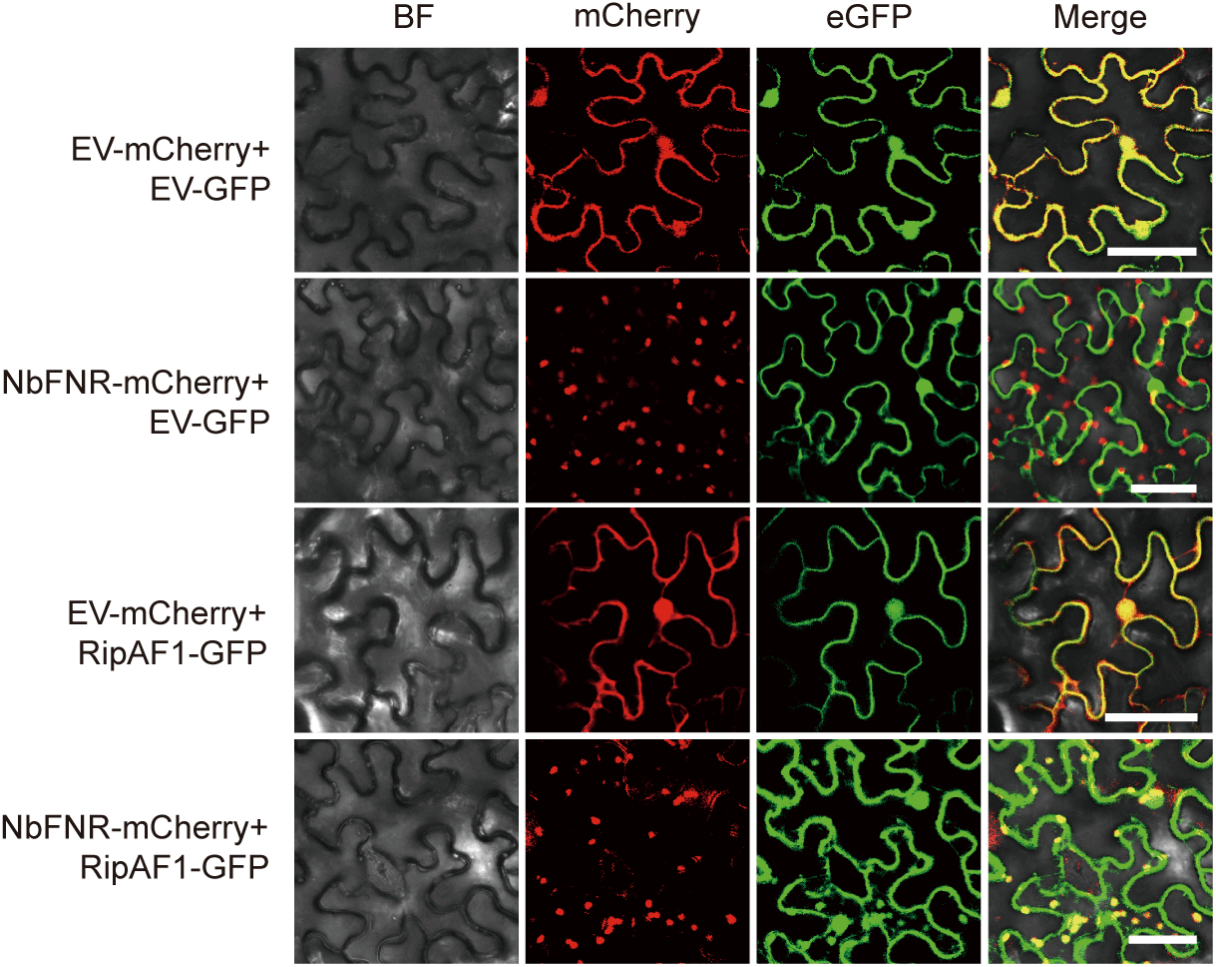
Subcellular localization analysis of RipAF1 and NbFNR. NbFNR-mCherry and RipAF1-GFP were co-expressed in *N. benthamiana* leaf and visualized by confocal microscopy. EV-GFP co-expressed with EV-mCherry, GFP co-expressed with NbFNR-mCherry and RipAF1 co-expressed with EV-mCherry were used as the controls. BF, bright field. Scale bar, 50 μm.

Previous studies have demonstrated that RipAF1 localizes to both the cell periphery and nucleus [1], while NbFNR is known to be chloroplast-localized. To examine potential co-localization of RipAF1 and NbFNR in plant cells, we generated fluorescent protein fusions (RipAF1-GFP and NbFNR-mCherry) and transiently expressed these constructs either individually or together in *N. benthamiana* leaves. Consistent with previous reports, RipAF1-GFP localized to the cell periphery and nucleus when expressed alone, whereas NbFNR-mCherry showed exclusive chloroplast localization (Fig 4). Strikingly, co-expression of both proteins resulted in the appearance of RipAF1-GFP fluorescence in chloroplasts (Fig 4), suggesting that NbFNR recruits RipAF1 to chloroplasts, thereby altering its subcellular distribution from the cell periphery and nucleus. This observed co-localization provides evidence for physical interaction between RipAF1 and NbFNR in chloroplast. To investigate the potential interaction between RipAF1 and NbFNR in chloroplasts, we conducted Co-IP assay using thylakoid membrane fractions isolated from *N. benthamiana* leaves co-expressing both proteins. Western blot analysis of the input fractions revealed strong accumulation of NbFNR in thylakoid membranes, whereas RipAF1 showed comparatively lower expression levels. Notably, following immunoprecipitation, NbFNR-myc was specifically detected in samples co-expressing RipAF1, but not in the control (S5 Fig). These results provide direct biochemical evidence for a physical interaction between RipAF1 and NbFNR within the chloroplast compartment.

### NbFNR positively regulates plant resistance against *R. solanacearum*

**Fig 5:**
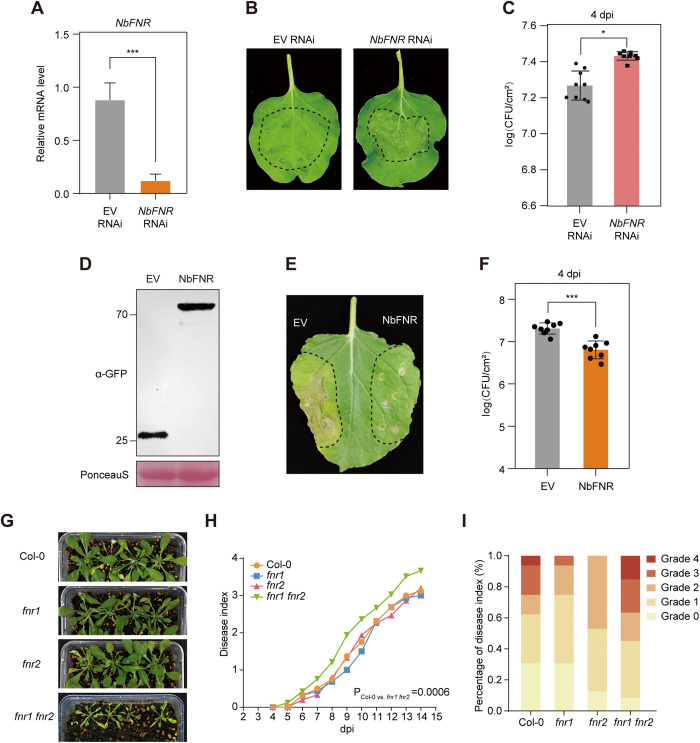
*NbFNR* positively regulates plant resistance against *R. solanacearum.* **(A)** Silencing of *NbFNR* in *N. benthamiana* by virus-induced gene silencing. *N. benthamiana* leaves were infiltrated with *A. tumefaciens* expressing the silencing constructs and the mRNA level of *NbFNR* was quantified at 2 weeks post infiltration. Data shown indicate mean ± SD (n = 3). **(B and C)** *R. solanacearum* growth in *NbFNR* silenced leaves. *NbFNR* silenced leaves or control leaves were infiltrated with *∆ripAF1* at 10^5^ CFU/ml. Leaf symptoms (**B**) were taken at 3 days post inoculation. Bacterial growth (**C**) was quantified at 4 days post inoculation. The images in panel (**B**) show one representative leaf from each treatment group (n = 9). Data shown in (**C**) indicate mean ± SD (n = 9); *p < 0.05 (Student’s *t*-*t*est); solid dots, individual biological replicates. **(D)** Over-expression of *NbFNR* in *N. benthamiana*. The NbFNR-GFP construct was expressed in *N. benthamiana* leaves and the protein level of NbFNR-GFP or the GFP control was determined by western blot with anti-GFP antibody. **(E and F)** *R. solanacearum* growth in *NbFNR* overexpressed leaves. *NbFNR* overexpressed leaves were infiltrated with *∆ripAF1* at 10^5^ CFU/ml. Leaf symptoms (**E**) were taken at 4 days post inoculation. Bacterial growth (**F**) was quantified at 4 days post inoculation. Data shown indicate mean ± SD (n = 9); ***p < 0.001 (Student’s *t*-tes*t*); solid dots, individual biological replicates. The experiment was repeated three times with similar results. (**G** to **I**) Soil-drenching inoculation assay in *fnr1*, *fnr2* and *fnr1 fnr2* double mutants. Four-week-old *A. thaliana* plants were inoculated with CQPS-1. **(G)** The disease phenotype of *fnr1*, *fnr2* and *fnr1 fnr2* at 10 dpi. **(H)** Disease index was recorded daily. Each value represents the mean disease index (n = 18). Statistics analysis was performed with Dunnett’s multiple comparison test. (**I**) indicates the disease index distribution at 9 dpi. Grade represents the disease index. The experiment was repeated three times with a similar result.

The conserved interaction between RipAF1 and FNR suggests that FNR may be involved in bacterial wilt resistance. To determine the role of FNR during *R. solanacearum* infection, we silenced *NbFNR* in *N. benthamiana* leaves using virus-induced gene silencing. The decrease of *NbFNR* mRNA was confirmed by quantitative real-time PCR (qPCR) (Fig 5A). *NbFNR* silenced leaves were inoculated with the *∆ripAF1* mutant strain, which retains virulent but carries a tetracycline-resistance gene for bacterial selective growth and quantification. Compared to an empty vector control, the silencing of *NbFNR* enhanced plant susceptibility to *R. solanacearum* and promoted bacterial replication (Fig 5B and 5C), suggesting that NbFNR functions as a positive regulator of plant resistance against *R. solanacearum*. To further validate this observation, we over-expressed a C-terminal GFP-tagged NbFNR in *N. benthamiana* leaves using *A. tumefaciens*-mediated transient gene expression, and NbFNR protein accumulation was validated by Western blot (Fig 5D). Over-expression of *NbFNR* reduced disease development and bacterial replication upon *R. solanacearum* inoculation (Fig 5E and 5F). To further validate the positive role of FNR in bacterial wilt resistance, we obtained *A. thaliana fnr1* and *fnr2* single mutants and generated *fnr1 fnr2* double mutant through genetic crossing (S6 Fig). While disease progression in *fnr1* and *fnr2* single mutants were comparable to wild-type plants, the *fnr1 fnr2* double mutant exhibited accelerated wilting symptoms following WT *R. solanacearum* infection (Fig 5G to 5I). This genetic evidence confirms that *AtFNR* contributes to bacterial wilt resistance, with functional redundancy between *FNR1* and *FNR2* isoforms.

Given that RipAF1 targets FNR to promote virulence, we investigated whether FNR is involved in RipAF1-mediated pathogenicity enhancement. We inoculated Col-0 and *fnr1 fnr2* mutant with either the WT strain or the *∆ripAF1*. The WT strain was more virulent than *∆ripAF1* in Col-0, while this virulence differential between WT and *∆ripAF1* was markedly attenuated in *fnr1 fnr2* (S7 Fig), suggesting a key role of *FNR1* and *FNR2* in RipAF1-mediated virulence potentiation. To further elucidate the functional interplay between NbFNR and RipAF1, we transiently overexpressed *NbFNR* in *N. benthamiana* leaves, followed by infiltration with either the WT strain or the Δ*ripAF1* mutant. Quantitative assessment revealed that *NbFNR* over-expression significantly attenuated disease symptoms, as evidenced by reduced leaf wilting and impaired bacterial proliferation compared to empty vector controls (S8 Fig). Notably, this suppression effect was more pronounced in plants inoculated with the Δ*ripAF1* strain than the WT strain (S8 Fig), indicating that RipAF1 partially counteracts NbFNR-mediated resistance during pathogen infection. These results indicate that RipAF1 targets FNR to promote *R. solanacearum* infection.

### RipAF1 decreases NADPH and ATP production

**Fig 6:**
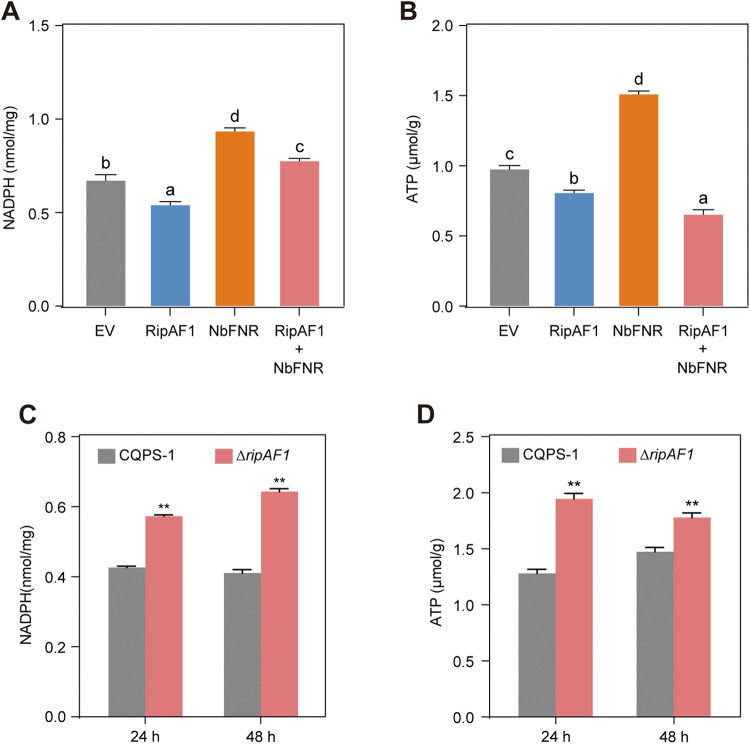
RipAF1 interferes with the activity of NbFNR and decreases the ATP accumulation. **(A and B)** NADPH (**A**) and ATP (**B**) content in *N. benthamiana* leaves after over-expression of RipAF1, NbFNR or co-expression of RipAF1 and NbFNR. *N. benthamiana* leaves were infiltrated with *A. tumefaciens* expressing RipAF1, NbFNR, or co-expression of RipAF1 and NbFNR. *A. tumefaciens* expressing GFP was used as the negative control. NADPH and ATP content were quantified at 2 days post infiltration. Data shown indicate mean ± SD (n = 3). Different letters indicate statistical difference between different samples (one-way ANOVA, *p* < 0.05). **(C and D)** NADPH (**C**) and ATP (**D**) content in *N. benthamiana* leaves after infiltration with *R. solanacearum* wild-type CQPS-1 or *∆ripAF1* knockout mutant. NADPH and ATP content were quantified at 24 and 48 hours post infiltration. Data shown indicate mean ± SD (n = 3); **p < 0.01 (Student’s *t*-tes*t*).

FNR in plants receives electrons from ferredoxin at the final step of the photosynthetic electron transport chain, and catalyzes the reaction from NADP^+^ to NADPH [43]. To confirm the role of NbFNR in NADPH production in our experimental system, we over-expressed *NbFNR* in *N. benthamiana* leaves and quantified NADPH content. Over-expression of *NbFNR* resulted in a significant increase in NADPH levels (Fig 6A), confirming the contribution of NbFNR to NADPH synthesis in *N. benthamiana* leaves. It is known that FNR is also involved in cyclic electron transfer around photosystem I, leading to ATP accumulation [33,44]. Therefore, we further measured ATP content after *NbFNR* over-expression. Compared to the empty GFP control, over-expression of *NbFNR* also significantly enhanced ATP accumulation (Fig 6B). Given the important role of ATP in the regulation of plant immunity [18], we hypothesized that RipAF1 manipulates host ATP levels through interaction with FNR. We first tested if RipAF1 interferes with the activity of NbFNR in NADPH and ATP accumulation. Expression of *RipAF1* in *N. benthamiana* leaves led to the decrease of NADPH and ATP levels (Fig 6A and 6B). When *NbFNR* was co-expressed with *RipAF1*, *NbFNR*-mediated NADPH and ATP accumulation was significantly suppressed (Fig 6A and 6B), demonstrating that RipAF1 inhibits the activity of NbFNR *in planta*. Notably, co-expression of *NbFNR* and *RipAF1* resulted in higher NADPH levels compared to the empty vector control (Fig 6A), due to the strong enhancing effect of NbFNR on NADPH production, which was only partially attenuated by RipAF1. In contrast, the ATP level in *NbFNR* and *RipAF1* co-expressed sample was lower than that in the empty vector control (Fig 6B). This reduction probably results from RipAF1 directly inhibiting NbFNR activity—thereby limiting ATP production—and indirectly constraining ATP generation via reduced NADPH availability. To further characterize RipAF1’s role in energy metabolism regulation, we quantified NADPH and ATP levels in *N. benthamiana* leaves inoculated with either the WT or Δ*ripAF1* at 24 and 48 hours post-inoculation. Comparative analysis revealed significantly reduced NADPH and ATP concentrations in WT-infected tissues compared to Δ*ripAF1*-infected controls at both time points (Fig 6C and 6D). Altogether, these results indicate that RipAF1 reduces the host NADPH and ATP contents.

### ATP treatment promotes plant resistance against *R. solanacearum*

**Fig 7:**
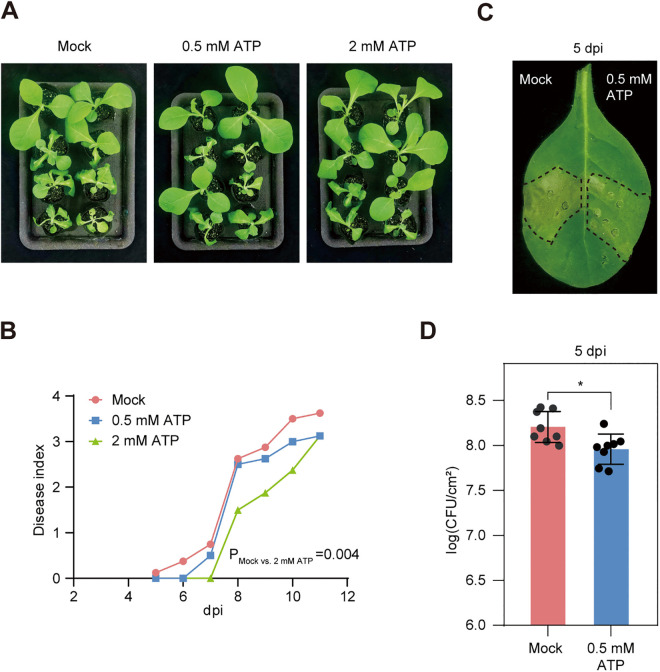
Exogeneous application of ATP enhances plant resistance against *R. solanacearum.* **(A and B)** Disease symptoms and disease index of tobacco seedlings pre-watered with ATP and inoculated with *R. solanacearum*. Pictures of bacterial wilt symptoms (**A**) were taken at 9 days post inoculation. Disease index (**B**) was rated daily using a scale of 0 to 4. Each value represents the mean disease index (n = 8). Statistics analysis was performed with Dunnett’s multiple comparison test. **(C and D)** *R. solanacearum* growth in ATP pretreated tobacco leaves. Leaves of four-week-old tobacco plants were infiltrated with 0.5 mM ATP or mock solution. One day later, the leaves were inoculated with *R. solanacearum* CQPS-1 at 10^5^ CFU/ml. Leaf symptoms (**C**) were taken at 5 days post inoculation. Bacterial growth (**D**) was quantified at 4 days post inoculation. Data shown indicate mean ± SD (n = 9); *p < 0.05 (Student’s *t*-*t*est); solid dots, individual biological replicates. The experiment was repeated three times with similar results.

ATP typically serves as an intracellular energy currency. ATP also serves as a DAMP molecule after being released into the extracellular space [10]. Given that RipAF1 reduces host ATP levels, we hypothesized that ATP plays an important role in *R. solanacearum* infection. To test this hypothesis, we pre-watered tobacco plants with solutions containing different concentrations of ATP. Treating tobacco roots with 2 mM of ATP by soil drenchning significantly delayed wilting symptom development caused by *R. solanacearum* (Fig 7A and 7B). We further treated tobacco leaves with a 0.5 mM ATP solution by needle-free syringe infiltration, and subsequently infiltrated with *R. solanacearum*. A lower ATP concentration was selected because ATP was delivered directly into the leaf tissue via syringe infiltration. Mock-treated leaves developed severe wilting symptoms at 5 days post inoculation, while ATP-treated leaves showed much less severe wilting symptoms (Fig 7C). Bacterial numbers were further quantified to determine the extent of infection. Leaves pretreated with ATP showed reduced bacterial titers compared with the mock treatment (Fig 7D). These data suggest that ATP induces defense reactions against *R. solanacearum*.

## Discussion

*R. solanacearum* typically encodes 45–75 different effector proteins per strain [45]. Although much progress has been made in determining the molecular functions of *R. solanacearum* effector proteins in the past decade [23], the virulence function and the host target(s) for most of the effectors remain to be identified. In this study, we demonstrated that RipAF1, one of the conserved type III effectors in *R. solanacearum*, targets host FNR to promote bacterial infection. We found that RipAF1 contributes to *R. solanacearum* virulence on tobacco, *N. benthamiana* and *A. thaliana*. In support of our observation, a previous study revealed that RipAF1 from the reference strain GMI1000 promotes bacterial fitness in eggplant [29]. Two recent studies tested the ability of RipAF1 in eliciting cell death and found that this effector failed to cause cell death in *Nicotiana* spp. [31,46]. Consistently, transient expression of RipAF1 in our work did not trigger any visible cell death in *N. benthamiana* (S9 Fig). However, RipAF1 from the *R. solanacearum* strain FJ1003 induced weak visual chlorosis and resistance in *N. benthamiana* [32]. Amino acid sequence alignment of RipAF1 between CQPS-1 and FJ1003 revealed complete sequence identity (S10 Fig), excluding protein sequence variation as the basis for their phenotypic differences. However, genomic analysis uncovered a significant organizational distinction: while RipAF1 is located on the megaplasmid in CQPS-1 (NZ_CP016915) and related strains including GMI1000 (AL646052), it is chromosomally encoded in FJ1003 (CP087278). This differential genomic localization suggests potential variations in RipAF1 expression dynamics during infection, which may contribute to the observed strain-specific pathogenicity differences. Another alternative explanation is that RipAF1 from strains CQPS-1 and FJ1003 may interact with other strain-specific factors, such as a divergent suite of type III effectors or PAMPs, which could alter how plant immunity is sensed or modulated. Furthermore, discrepancies in growth conditions, plant health, and *A. tumefaciens* infiltration parameters may also explain the phenotypic differences observed in RipAF1.

FNR is a conserved flavoenzyme in plants. Although its physiological role in mediating the electron transfer from reduced ferredoxin to NADP^+^ has been well studied [47], the biological functions of FNR are relatively less known. *FNR* genes are involved in abiotic stress responses, such as drought stress [48]. A recent study reported the positive role of FNR in assisting bamboo mosaic virus accumulation in *N. benthamiana* [49]. Our results revealed that transient expression of *NbFNR* enhanced plant resistance against *R. solanacearum* (Fig 5E and 5F). Together with our work, it highlights that FNR has important functions during plant-pathogen interactions. However, our assay was based on transient expression of *NbFNR*, which has limitation compared to stable transgenic plants. Generating stable transgenic lines in *N. benthamiana* in the future will further strengthen the role of *NbFNR* in plant resistance against pathogens. Nevertheless, the molecular mechanism of FNR-mediated plant-pathogen interaction remains to be determined. Expression of plant FNR in *Escherichia coli* has been demonstrated to restore the oxidative tolerance of a mutant strain [50]. The activity of FNR might conserved across kingdoms, and suggests that plant FNR is also involved in oxidative stress responses. We identified FNR as the host target of RipAF1. It has been shown that expression of RipAF1 delayed ROS burst triggered by the immune elicitor flg22 [31]. Consistently, our results showed that RipAF1 could suppress flg22-induced ROS production (Fig 2C). Therefore, beyond its role in modulating ATP homeostasis to promote plant resistance, FNR may also regulate ROS levels to enhance defense against *R. solanacearum*, a mechanism that is possibly used by the bacterial effector RipAF1. The chloroplast-localized interaction between RipAF1 and FNR indicates that RipAF1 might function at later stages of infection when *R. solanacearum* starts to colonize leaf mesophyll and vascular tissues (S5 Fig).

ATP is mainly produced intracellularly. Upon cell damage, ATP is released from cells and acts as a DAMP signal [10]. Our results showed that RipAF1 interacts with FNR in chloroplast, it is likely that RipAF1 reduces ATP production in the chloroplast. Notably, chloroplast-produced ATP is primarily consumed locally, while ATP produced from mitochondrial can supply energy when chloroplast ATP is limited. A reduction in chloroplast ATP could potentially indirectly reduce mitochondrial and thus total ATP levels. However, the chloroplast synthesized ATP might be released to the apoplast when a cell is damaged, where ATP is perceived and induces downstream signaling events. On the other hand, as a pathogenic bacterium, *R. solanacearum* colonization causes plant cell damage at the colonization site [51]. This may lead to the rapid release of ATP to the extracellular space, where it may function as a DAMP activating plant immunity. Therefore, it is possible that RipAF1-mediated ATP reduction in chloroplast could potentially reduce intracellular ATP and affects the extracellular ATP after cell damage. Reduction of plant ATP concentration suppresses immunity activation and thus promotes *R. solanacearum* infection. Mitochondria and the cytosol serve as major sources of cellular ATP, which can be released into the extracellular space as DAMPs during cellular injury. While mitochondrial and cytosolic ATP levels may partially mask the impact of chloroplast-derived ATP fluctuations on total cellular ATP content, a decrease in chloroplast ATP production typically leads to an overall reduction in total ATP levels. In addition to its role as an extracellular DAMP molecule, an alternative mechanism may involve intracellular ATP serving as an energy currency to fuel plant defense responses. The interaction between RipAF1 and FNR, which compromises chloroplast ATP production, could consequently deplete the energy supply required for effective defense activation, thereby facilitating pathogen infection.

Extracellular ATP treatment induces expression of plant defense associated genes [19]. Some of the ATP responsive genes are dependent on defense hormone signaling pathways such as jasmonate or ethylene, while others are independent on classic hormone signaling pathways [19]. It has been shown that ATP pretreatment in *A. thaliana* leaves increases plant resistance against the necrotrophic fungus *Botrytis cinerea* by activating the intracellular jasmonate signaling [18]. Application of ATP also induces resistance to the bacterial pathogen *P. syringae* through rapid closure of the leaf stomata [13]. Our results revealed that ATP pretreatment positively regulates plant resistance against *R. solanacearum*. These findings consistently support the positive effect of ATP in regulation of plant immunity. Of note, the ATP receptor P2K1 is involved in ATP-mediated resistance against *B. cinerea* and *P. syringae* [13,17,18]. Heterologous expression of the *A. thaliana* ATP receptor enhances resistance to *P. infestans* in solanaceous plants [15]. It remains to be tested if ATP-mediated resistance to *R. solanacearum* is also dependent on DORN. On the other hand, residues from 155 to 338 of RipAF1 possessing an ADP-ribosyltransferase domain, which can ribosylate FBN1 protein to regulate hormone homeostasis [32]. Therefore, FNR may also be post-translationally regulated, thus affecting protein stability and regulating ATP production.

**Fig 8:**
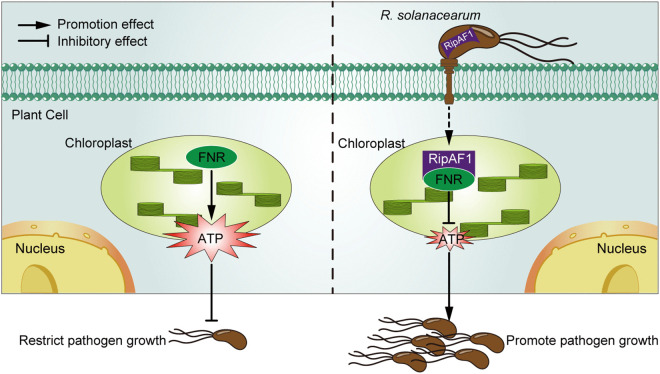
A simplified working model of RipAF1. Under normal conditions, FNR is involved in ATP production, which positively regulates plant resistance and therefore restricts pathogen growth. During an infection, *R. solanacearum* uses the needle-like type III secretion system to translocate RipAF1 into host cells. RipAF1 interacts with FNR in chloroplast and decreases host ATP levels promoting bacterial infection. The arrow and the blunt arrow represent the promoting and inhibitory effects, respectively.

In summary, we establish RipAF1 as a virulence effector of *R. solanacearum* that physically interacts with FNR, which is involved in ATP production and plant resistance against *R. solanacearum* (Fig 8). We further demonstrated the key role of ATP in resistance to *R. solanacearum*. Given that ATP is a universal energy currency throughout the animal and plant kingdoms and extracellular ATP acts as a DAMP signaling molecule, host ATP levels could represent an important hub that is targeted by animal and plant pathogenic bacteria for their own benefit.

## Materials and methods

### Plant materials and growth conditions

The *A. thaliana* Columbia-0 (Col-0) was used as wild type (WT). T-DNA insertion mutants of *fnr1* (*SALK_067668*) and *fnr2* (*SALK_017328*) were obtained from AraShare (https://www.arashare.cn/index/). *fnr1 fnr2* was obtained through crossing *fnr1* with *fnr2*. Seeds were germinated on ½ Murashige & Skoog (MS) medium, then transferred to the soil. *A. thaliana* were grown at 22°C under long day conditions (16-h light/8-h dark cycles). *N. benthamiana* and *N. tabacum* seeds were sown in soil and transplanted into new pots after germination. Seedlings were grown in a growth chamber with 12-h light/12-h dark photoperiod at 22°C.

### *R. solanacearum* strains and growth conditions

The *R. solanacearum* wild-type strain CQPS-1 (WT) was kindly provided by Prof. Wei Ding (Southwest University). The *∆ripAF1* mutant strain and the *ripAF1*^*+*^ complementation strain were generated as previously described [52]. Briefly, for the generation of the *∆ripAF1* mutant, the upstream fragment and downstream fragment (≈ 750 bp) flanking *RipAF1* coding sequence were PCR amplified and cloned into LI backbone via *BpiI* cut-ligation reaction. LI vectors containing the upstream, the tetracycline gene and the downstream were further assembled into LII destination vector via *BsaI* cut-ligation, resulting the LII knockout construct. Subsequently, the LII knockout vector was linearized, purified and transformed into the WT strain as described previously [53]. The *∆ripAF1* mutant strain was first selected on tetracycline resistant plate and then confirmed by PCR. To assemble the complementation construct, the coding sequence of *RipAF1* together with its native promoter (186 bp) was PCR amplified and cloned into the LI backbone. The LI pRipAF1_RipAF1 module was further cloned into a LII backbone with a myc epitope tag and a gentamycin selection marker. The LII pRipAF1_RipAF1_myc_gent vector was combined into the LIII vector that contains the recombinant regions for integration of the insertion into the *R. solanacearum* genome [53]. Transformation of the complementation construct into the *∆ripAF1* mutant was the same as for the knockout. The *RipAF1*^*+*^ complementation strain was confirmed by both resistance selection and PCR. *R. solanacearum* strains were grown in B medium (Peptone, 10 g/L; Tryptone, 1 g/L; Yeast extract, 1 g/L; Glucose 5 g/L; Agar, 15 g/L; Triphenyl Tetrazolium Chloride, 20 mg/L) at 28°C. When needed, antibiotics were used with the following concentrations: ampicillin, 50 mg/ml; gentamycin, 15 mg/ml; kanamycin, 50 mg/ml; spectinomycin, 100 mg/ml; tetracycline, 10 mg/ml. All primers used in this study were listed in S2 Table.

### Pathogenicity assay

Four-week-old *N. benthamiana* or *N. tabacum* or *A. thaliana* plants were soil drenching inoculated with 10 ml of OD_600_ = 0.2 bacterial suspension. Inoculated plants were kept in a growth chamber with 12 h light/12 h dark photoperiod, 28°C and the disease symptoms of each plant were recorded daily using a disease index scale of 0–4 (0, no leaf wilted; 1, 1% to 25% leaves wilted; 2, 26% to 50% leaves wilted; 3, 51% to 75% leaves wilted; 4, 76% to 100% leaves wilted). For bacterial quantification in *N. benthamiana*, leaves of 4- to 5-week-old plants were first infiltrated with *A. tumefaciens* expressing the gene of interest. Infiltrated areas were further infiltrated with *R. solanacearum* at 10^5^ CFU/ml. Leaf discs were harvested at 2 or 4 dpi and ground in sterile water. Series of dilutions were plated out on B plates with appropriate antibiotic and incubated at 28°C for 36–48 h. For ATP pre-treatment, 0.1 M of ATP (A2383, Sigma-Aldrich) stock solution was prepared in 2 mM of MES buffer (pH = 5.7) and filter sterilized. The stock solution was diluted to 0.5 mM or 2 mM for pre-treatment. Working solution of ATP was either directly poured into the rhizosphere of the seedlings (10 ml per seedling) or infiltrated into leaves of 3- to 4-week-old tobacco plants.

### ROS assay

Leaves of 4- to 5-week-old *N. benthamiana* plants were infiltrated with *A. tumefaciens* expressing GFP or RipAF1-GFP fusion protein. Leaf discs were harvested from infiltrated areas at 2 dpi with a hole punch (diameter 2 mm), placed in a 96-well plate with each well containing 100 µl of sterile water, and incubated overnight avoid from light. On the second day, the sterile water was discarded and 100 µl of solution containing 17 μg/ml Luminol L-012 (143556-24-5, Sigma-Aldrich), 10 μg/ml horseradish peroxidase (HRP, P8375, Sigma-Aldrich), and 100 nm flg22 (pH = 5.8), were added to each well. Luminescence was measured immediately using a Fluoroskan Ascent FL microplate fluorometer and luminometer (Thermo Scientific). ROS production is reported as an increase in photon counts or a sum of total photon counts.

### MAPK assay

*A. tumefaciens*-mediated RipAF1 transient expression in *N. benthamiana* was the same as for ROS assay. At 2 dpi, flg22 solution (500 nM) was infiltrated into leaves expressing GFP or RipAF1-GFP fusion protein. Leaf samples were harvested at 0 min, 5 min and 15 min after flg22 treatment. Total proteins were extracted with the extraction buffer RIPA (EA0004; SparkJade) supplemented with 1 mM NaF and 1 mM Na_3_VO_4_, two commonly used phosphatase inhibitors, and collected after centrifugation for 5 min at 4°C. The protein samples with SDS buffer were boiled at 95°C for 10 min, ensure that the proteins in the sample are fully dissolved and evenly distributed in the buffer. The mixture was loaded on SDS-PAGE and western blot was carried out with anti-pMAPK (a phospho-p38 MAPK (Thr180/ Tyr182)) (#9212, Cell Signaling Technology). The Ponceau S is stained by Ponceau S staining solution (P0022, Beyotime).The membrane is incubated in staining solution at room temperature for 5–10 min. Then the membrane was washed in deionized water for 1–5 min until the red band is visible.

### Large-scale immunoprecipitation and LC-MS/MS analysis

*A. tumefaciens* expressing either the GFP control or the RipAF1-GFP fusion protein were infiltrated into leaves of 4- to 5-week-old *N. benthamiana* plants at OD_600_ = 0.4. Two grams of infiltrated leaves were harvested at 2–3 dpi and frozen in liquid nitrogen immediately. The tissue was homogenized in protein lysis buffer (10 mM Tris-HCl, PH = 7.5, 150 mM NaCl, 0.5 mM EDTA, 5% Glycerol, 1% Triton X-100, 1 × cocktail [B15001; Selleck], 1 mM PMSF), incubated at 4°C for 1 h and centrifuged at 6, 000 rpm for 10 min. The supernatant was transferred to a new tube. 40 µl of anti-GFP Magarose Beads (SM038001; SMART Lifescience) were added to the protein extract and incubated overnight at 4°C with constant and mild rotation. The sample was centrifuged at 4°C to remove the supernatant. GFP-trap beads were washed three times with ice cold wash buffer (50 mM Tris-HCl [PH = 7.5], 150 mM NaCl,1 mM EDTA, 5% Glycerol, 1 × cocktail [B15001; Selleck], 1 mM PMSF, 0.1% Triton X-100). SDS were added to the washed beads to a final concentration of 1 × and the mixture were boiled at 95°C for 10 min. Immunoprecipitated proteins were separated by SDS-PAGE. Each lane from the gel containing all the protein was cut, proteins were digested in gel with trypsin and subjected to Mass Spectrometric analysis to identify interacting proteins (oebiotech). For spectral searches, the database of Niben101_annotation.tobacco_proteins from Sol Genomics was used.

### Split-LUC assay

Split-LUC assay was performed as previously described with minor modifications [54]. Briefly, RipAF1 was cloned into C-LUC and FNRs were cloned into N-LUC, respectively. Plasmids were transformed into *A. tumefaciens* GV3101. *A. tumefaciens* strains containing the N-LUC and the C-LUC were equally mixed and infiltrated into leaves of 4- to 5-week-old *N. benthamiana* plants. At 36–48 hpi, the *A. tumefaciens* infiltrated areas were infiltrated with 0.1 mM luciferin (C3654, APExBIO) and kept in the dark for 5 min. The images were taken with the plant living imaging system (NEWTON7.0 Bio plus, VILBER).

### *In vitro* pull-down assay

The coding sequence of *RipAF1* and *NbFNR* were PCR amplified and cloned into pGEX-4T-1 and pET32a, respectively. The vectors were transformed into *E. coli* stain BL21. Protein expression was induced with 0.5 mM Isopropyl β-D-Thiogalactoside at 16°C. *E. coli* cells expressing GST, RipAF1-GST and NbFNR-His were collected by centrifugation. Bacterial pellets were resuspended in GST lysis buffer (150 mM NaCl, 50 mM Tris-HCl [pH = 7.5]) or His lysis buffer (300 mM NaCl, 50 mM Tris-HCl [pH = 7.5], 10 mM Imidazole) supplemented with 1 × solution of the protease inhibitor PMSF and sonicated. The supernatant was centrifuged at 7, 500 rpm. After centrifugation, the supernatant was mixed with prewashed Glutathione Beads (SA008025; SMART Lifescience) and Ni-NTA Sefinose Resin (C600033; BBI) and incubated overnight at 4°C. Subsequently, the beads were washed with GST wash buffer (300 mM NaCl, 50 mM Tris-HCl [pH = 7.5]) or His wash buffer (300 mM NaCl, 50 mM Tris-HCl [pH = 7.5], 20 mM Imidazole, pH 8.0) for three times. Purified fusion proteins were eluted with GST elution buffer (300 mM NaCl, 50 mM Tris-HCl [pH = 7.5], 20 mM Glutathione) or His elution buffer (300 mM NaCl, 50 mM Tris-HCl [pH = 7.5], 250 mM Imidazole). For GST pull-down assay, the agarose beads were prewashed with GST wash buffer. The GST protein and RipAF1-GST fusion protein were mixed with NbFNR-His fusion protein, respectively and incubated with the prewashed agarose beads in GST-binding buffer overnight at 4°C. The incubation mixture was pelleted by centrifugation and the precipitate was washed five times with GST wash buffer containing 0.5% Triton X-100. The pellet was then eluted in 50 µl of GST elution buffer. After centrifugation, the eluate was mixed with SDS loading buffer, boiled and analyzed using SDS-PAGE.

### Co-IP assay

Leaves of 4- to 5-week-old *N. benthamiana* plants were co-infiltrated with *A. tumefaciens* expressing RipAF1-GFP and FNR-Myc fusion proteins. Leaves were harvested at 2–3 dpi and ground in liquid nitrogen. Total proteins were extracted using the extraction buffer (10 mM Tris-HCl [PH = 7.5], 150 mM NaCl, 0.5 mM EDTA, 5% Glycerol, 1% Triton X-100, 1 × protease inhibitor cocktail [B15001; Selleck], 1 mM PMSF). The extracts were centrifuged several times at 4°C until there was no precipitation. The supernatant was immunoprecipitated with 20 µl of anti-GFP Magarose Beads (SM038001; SMART Lifescience) overnight at 4°C. After centrifugation, beads were washed three times with wash buffer (50 mM Tris-HCl, PH = 7.5, 150 mM NaCl, 1 mM EDTA, 5% Glycerol, 0.1% Triton X-100, 1 × protease inhibitor cocktail [B15001; Selleck], 1 mM PMSF). The immunoprecipitated proteins were eluted from the beads by adding SDS and boiling at 95°C for 10 min. Proteins with GFP or Myc tags were detected by anti-GFP or anti-Myc antibody, respectively.

### Chloroplast protein extraction

Chloroplast proteins from *N. benthamiana* leaves were extracted as previously described with some modifications [53]. Leaves were ground with ice-cold CI buffer (400 mM sucrose, 5 mM MgCl_2_, 10 mM KCl, 0.1% BSA, 1 × protease inhibitor cocktail [B15001; Selleck]), and filtered the homogenate with four layers of cheesecloth. Then centrifuge the samples at 3,000 × g for 15 min at 4°C to remove the supernatant. Resuspend the crude chloroplasts with cold CI buffer and carefully pipette it onto the Percoll gradient (0.9 ml Percoll, 0.1 ml sucrose, 16,000 × g for 30 min with soft braking). Carefully connect the green band into new eppendrof tube and wash it twice with CI buffer. Ensure all materials strictly chilled below 4°C throughout the procedure to safeguard chloroplast integrity and activity.

### Subcellular localization analysis

The subcellular localization of each fluorescence fusion construct was analyzed using a confocal laser microscope (Leica STELLARIS 5; Leica). This analysis was carried out 48 hours after transient expression in *N. benthamiana*. RipAF1 was inserted into the pCAMBIA1300-GFP vector, and NbFNR was cloned into pCAMBIA1300-mCherry, resulting in the formation of RipAF1-GFP and NbFNR-mCherry fusion constructs. The observations of NbFNR-mCherry and RipAF1-GFP were conducted at excitation wavelengths of 488 nm and 514 nm, respectively, with corresponding bandpass emission filters of 470–550 nm and 530–560 nm.

### Virus-induced gene silencing

To silence the *NbFNR* in *N. benthamiana*, a 449-bp fragment of *NbFNR* was amplified from cDNA of *N. benthamiana* leaf infiltrated with *A. tumefaciens* expressing RipAF1-GFP. The PCR product was cloned into pTRV2 vector to create the silencing construct pTRV2:NbFNR. *A. tumefaciens* containing pTRV1 and pTRV2 empty vector or pTRV2:NbFNR were mixed in a 1:1 ratio and infiltrated into leaves of 2- to 3-week-old *N. benthamiana* plants. The silencing efficiency was determined by qRT-PCR at 2 weeks post infiltration, as described below.

### RNA extraction and quantitative real-time PCR

Total RNA was isolated from plant tissues with the RNAprep pure Plant Kit (DP432; Tiangen) according to the manufacturer’s instructions. The quality and concentration of total RNA was checked by nanodrop. cDNA was synthesized from 500 ng of total RNA with the HiScript II 1st Strand cDNA Synthesis Kit (R212; Vazyme) according to the protocol provided by the company. Quantitative Real-Time PCR (qPCR) was carried out in a 10 µl reactions (CFX96; BioRad), each reaction containing 1 µl of 1–10 diluted cDNA, 1 pmol of forward and reverse primer, 5 µl of ChamQ Universal SYBR qPCR Master Mix (Q711-02; Vazyme). The PP2A gene from *N. benthamiana* was used as a reference gene for the internal normalization [25]. Relative expression of genes was calculated with 2^-∆∆CT.^

### NADPH quantification

NADPH content was measured using a Coenzyme II (NADP/NADPH) content test kit (BC5200; Solarbio) according to the manufacturer’s instructions. Briefly, leaves of 4-week-old *N. benthamiana* plants were infiltrated with *A. tumefaciens* expressing indicated constructs. 0.1 g of fresh leaves were ground in ice-cooled mortar with 0.5 ml alkaline extraction buffer, boiled for 5 min and then cooled in ice bath. The samples were centrifuged at 10,000 × g for 10 min at 4°C, 200 µL of the supernatant were collected, and mixed with an equal volume of acidic extraction buffer, followed by centrifuging at 10,000 × g for 10 min at 4°C. The upper aqueous phase were collected carefully and kept on ice until analysis. The sample were mixed with the reagent described in the instruction manual and the absorbance were measured at 570 nm. The NADPH content was calculated according to the instructions provided by the test kit (BC5200; Solarbio).

### ATP quantification

ATP content in leaf tissue was quantified using an ATP content test kit (BC0300; Solarbio) according to the manufacturer’s instructions. 0.1 g of fresh leaves were ground in ice-cooled mortar with 1 ml extraction buffer, followed by centrifuging at 10,000 × g for 10 min at 4°C. The supernatant were collected in a new eppendrof tube. 500 μL of ice-cold chloroform were added and mixed thoroughly by vigorous vortexing, followed by centrifuging at 10,000 × g for 3 min at 4°C. The upper aqueous phase were collected carefully and kept on ice until analysis. After adding the sample and the reagent described in the instruction manual to the quartz cuvette respectively. The absorbance value were measured at 340 nm at 10 seconds after mixing. The ATP content was calculated according to the instructions provided by the test kit (BC0300; Solarbio).

### Statistical analysis

Statistical analysis was performed in GraphPad Prism 8.0.1. Specifically, Dunnett’s multiple comparison test was used to compare the significant difference between the control and treatments in the disease index assay. The *p*-value was calculated on a combined analysis of the average diseases index over time. One-way ANOVA followed by Tukey’s HSD test (SPSS Statistics 27) was used for statistical analysis among multiple groups. The Student’s *t*-test was used to compare the significance between two groups.

## Supporting information

S1 Fig Generation of RipAF1 mutant and complementation strains. (**A** and **B**) Schematic display of the generation of the *∆ripAF1* mutant and *RipAF1* complementation strains. (**A**) The resistance selection marker gene tetracycline (Tet) was used to replace the coding sequence of *RipAF1*. The numbers above the genes indicate the PCR product size of wild-type strain (WT) and *∆ripAF1* mutant strain, respectively. (**B**) Native promoter-driven *RipAF1* was integrated between nucleotides 203336 and 203337 of the *∆ripAF1* mutant genome. A forward primer from the upstream of the integration site and a reverse primer from the coding sequence of *RipAF1* was used for PCR validation of the complementation. (**C** and **D**) PCR confirmation of the *∆ripAF1* mutant and *RipAF1* complementation strains. (**C**) Gel picture showing the PCR products amplified from the WT and *∆ripAF1* mutant strains. The numbers at the left side indicate the marker size in base pairs. (**D**) Gel picture showing the PCR products amplified from the *∆ripAF1* mutant and *ripAF1* complementation strains (*ripAF1*^*+*^).
(TIF)

S2 Fig Immunoprecipitation and LC-MS/MS of RipAF1-GFP fusion protein. (**A** and **B**) Western blot analysis of RipAF1-GFP or GFP proteins before (**A**) and after (**B**) immunoprecipitation. (**A**) RipAF1-GFP or GFP was expressed in *N. benthamiana* leaves and samples were harvested at 2 days post inoculation. Immunoblots were performed with an anti-GFP antibody. (**B**) Total proteins from (**A**) were immunoprecipitated with anti-GFP Magnetic beads. Immunoprecipitated proteins were further confirmed by western blot with anti-GFP antibody. Two replicates were performed for RipAF1-GFP (RipAF1 #1, RipAF1 #2) and one replicate was performed for GFP vector control. (**C**) LC-MS/MS of RipAF1-GFP immunoprecipitated protein fractions identified NbFNR as a top candidate interactor of RipAF1. The picture includes the number of unique peptides that mapped to NbFNR, the sequence of the detected peptides and the relative position of the detected peptides.
(TIF)

S3 Fig Expression and purification of GST tagged RipAF1 and His tagged NbFNR fusion proteins. RipAF1-GST or NbFNR-His constructs were transformed into *E. coli* BL21. Protein expression was induced with 0.5 mM IPTG. The gel picture shows the total protein before induction, after induction and after purification. B, before IPTG induction; A, after IPTG induction; P, after GST purification. The lane of GST indicates the purified GST empty vector.
(TIF)

S4 Fig Phylogenetic tree analysis of FNR. Phylogenetic analysis of FNR from *R. solanacearum* host plants. MEGAX software was used to construct the phylogenetic tree using neighbor-joining methods. The bootstrap values from 10000 replications are indicated on the branch.
(TIF)

S5 Fig The interaction between RipAF1 and NbFNR in chloroplasts by co-immunoprecipitation assay. RipAF1-GFP and NbFNR-Myc were co-expressed in *N. benthamiana* leaves and samples were harvested at 3 days post inoculation. A chloroplast localized GFP was used as negative control. Chloroplasts protein was extracted and immunoprecipitated with anti-GFP magnetic beads. Immunoblots were performed with anti-GFP and anti-Myc antibodies, respectively.
(TIF)

S6 Fig Mutations identification of *fnr1* and *fnr2.* Identification of the T-DNA insertion mutants by Tri-Primer PCR method. LP and RP stand for left and right genomic primers. LB for left border primer of the T-DNA insertion. Two paired PCR reactions, LP + RP and LB + RP, were established. In the LP + RP reaction, a PCR product was obtained for wild-type or heterozygous lines, while no product (blank) was observed for homozygous mutant lines. Conversely, in the LB + RP reaction, a distinct band was detected for both homozygous mutant and heterozygous lines.
(TIF)

S7 Fig The disease phenotype of CQPS-1 or *∆ripAF1* in Col-0 and *fnr1 fnr2* mutant. (**A** to **D**) Soil-drenching inoculation of *R. solanacearum* wild-type strain CQPS-1 or *∆ripAF1* in Col-0 and *fnr1 fnr2* mutant. (**A** and **C**) Disease index were rated daily. Each value represents the mean disease index (n = 18). Statistics analysis was performed with Student’s *t*-test. (**B** and **D**) indicate the disease index distribution at 9 dpi. Grade represents the disease index. The experiment was repeated three times with a similar result.
(TIF)

S8 Fig The *in planta* growth of CQPS-1 or *∆ripAF1* in *NbFNR* overexpressed leaves. *NbFNR* overexpressed leaves were infiltrated with either CQPS-1 or *∆ripAF1* at 10^5^ CFU/ml. Leaf symptoms (**A** and **C**) were taken at 6 days post inoculation. Bacterial growth (**B** and **D**) was quantified at 4 days post inoculation. Data shown indicate mean ± SD (n = 9); **p < 0.01; ***p < 0.001 (Student’s *t*-test); solid dots, individual biological replicates. The experiment was repeated three times with similar results.
(TIF)

S9 Fig Transient over-expression of *RipAF1* does not cause necrosis. GFP (EV) or *RipAF1* was overexpressed in *N. benthamiana* leaves by *A. tumefaciens*-mediated transient gene expression. Leaf symptoms were taken at 2 days post inoculation.
(TIF)

S10 Fig Amino acid comparison of RipAF1 among CQPS-1, FJ1003 and GMI1000. RipAF1 from CQPS-1, FJ1003 and GMI1000 were aligned with the online multiple sequence alignment website (https://espript.ibcp.fr/ESPript/cgi-bin/ESPript.cgi).
(TIF)

S1 Table Candidate interactors of RipAF1 identified by IP-MS analysis. (XLS)

S2 Table Primers used in this study. (DOCX)
