# Supplementary material for: A bacterial effector protein targets plant ferredoxin-NADP+ reductase to promote infection
Source: PLoS Pathog. 2025 Oct 30;21(10):e1013664. doi: 10.1371/journal.ppat.1013664 (PMC12591422; doi:10.1371/journal.ppat.1013664)
Supplement: S2 Table — (DOCX) [file ppat.1013664.s012.docx]

**S2 Table. Primers used in this study.**

| Primer | sequence（5’-3’） |
| --- | --- |
| RipAF1 UP F | TTGAAGACTGTACGGGTCTCGCACCCTGGCCTGGAATTCCGCG |
| RipAF1 UP R | AAGAAGACGTCAGAGGTCTCGCTCAGCTGTACCTGCTCGATTGGG |
| RipAF1 DOWN F | TTGAAGACTGTACGGGTCTCGTGTCGGTGGACGCGGTTGCGGT |
| RipAF1 DOWN R | AAGAAGACGTCAGAGGTCTCGCCTTCCGCCGGGCGCAGCTTCCTG |
| PCR-F | TGAATGGATTTGACAAGCGTGC |
| PCR-R | ACTCGATGCTGGCGCTGG |
| RipAF1-GFP-F | TGACCTCGAGACTAGTATGGGTTTGCCACGGATCC |
| RipAF1-GFP-R | TCCTCTAGAGGATCCTCGCGTTGACGTGGACGCCT |
| Nb-FNR-Myc-F | GACCTCGAGACTAGTATGGCTGCTGCAGTAAGTGCT |
| Nb-FNR-Myc-R | TCGCACCATACTAGTGTAGACTTCAACATTCCATTGCT |
| Nb-FNR-GFP-F | TGACCTCGAGACTAGTATGGGTTTGCCACGGATCC |
| Nb-FNR-GFP-R | TCCTCTAGAGGATCCGTAGACTTCAACATTCCATTGCT |
| Nb-FNR-N-LUC-F | GGACGAGCTCGGTACCCATGGCTGCTGCAGTAAGTGCT |
| Nb-FNR-N-LUC-R | TACGAGATCTGGTCGACGTAGACTTCAACATTCCATTGCT |
| 32a-Nb-FNR-His-F | GCTGATATCGGATCCATGGCTGCTGCAGTAAGTGCT |
| 32a-Nb-FNR-His-R | ACGGAGCTCGAATTCGTAGACTTCAACATTCCATTGCT |
| Nb-FNR-mCherry-F | GGTGGAAGTGGTGGATCCATGGGTTTGCCACGGATCC |
| Nb-FNR-mCherry-R | TCCAGATCCACCGTCGACTCGCGTTGACGTGGACGCCT |
| RipAC-N-LUC-F | GGACGAGCTCGGTACCCATGCCTATCCTTCCACGCCT |
| RipAC-N-LUC-R | TACGAGATCTGGTCGACACGCTGCCTCGACGGACTT |
| Nb-SGT1-C-LUC-F | CGTCCCGGGGCGGTACCATGGCGTCCGATCTGGAGATT |
| Nb-SGT1-C-LUC-R | AAGCTCTGCAGGTCGACCTAGATTTCCCATTTCTTCAGCT |
| RNAi-Nb-FNR-F | ACGACAAGACCCTGCAGTGCTGCAGTTTCTCTTCCATCA |
| RNAi-Nb-FNR-R | GGAGAAGAGCCCTGCAGAGTCGCCGAAGTCACCAAG |
| QRNAi-Nb-FNR-F | GTCCACCTCTTTTCCCACCA |
| QRNAi-Nb-FNR-R | GGCACTGCTAGCAATGGAGT |
| qNb-PP2A-F | GCATATCATTCCTCAGGTCTTGG |
| qNb-PP2A-R | GGTGCAAGCAATGAAATCGC |
| pyy13-F | GTTCAGGCGGTTCTTGTGTGTCA |
| Sl-FNR-N-Luc-F | GGACGAGCTCGGTACCCATGGCTACTGCAGTAAGTGCT |
| Sl-FNR-N-Luc-R | TACGAGATCTGGTCGACGTAAACTTCAACATTCCATTG |
| At-FNR1 -N-Luc-F | GGACGAGCTCGGTACCCATGGCTGCTGCTATAAGTGCT |
| At-FNR1 -N-Luc-R | TACGAGATCTGGTCGACGTAGACTTCAACATTCCACT |
| At-FNR2 -N-Luc-F | GGACGAGCTCGGTACCCATGGCGACTACCATGAATGCT |
| At-FNR2 -N-Luc-R | TACGAGATCTGGTCGACGTAGACTTCAACGTTCCATTG |
| 4T-1-RipAF1-F | GTTCCGCGTGGATCCATGGGTTTGCCACGGATCC |
| 4T-1-RipAF1-R | TCGACCCGGGAATTCTCGCGTTGACGTGGACGCCTCC |
| RipAF1-C-LUC-F | CGTCCCGGGGCGGTACCATGGGTTTGCCACGGATCC |
| RipAF1-C-LUC-R | AAGCTCTGCAGGTCGACTCGCGTTGACGTGGACGCCTCC |
| At-fnr1-LP | AGCGAGACCATAATGTCATCG |
| At-fnr1-RP | ACATTGTCTTCACCACCGAAG |
| At-fnr2-LP | AGCACCCTTTTAAACACACCC |
| At-fnr2-RP | ATAGTTCAGGTGATGCAACCG |
| LB1.3 | ATTTTGCCGATITCGGAAC |
